# Strategies to attenuate maladaptive inflammatory response associated with cardiopulmonary bypass

**DOI:** 10.3389/fsurg.2024.1224068

**Published:** 2024-07-03

**Authors:** Debolina Banerjee, Jun Feng, Frank W. Sellke

**Affiliations:** Division of Cardiothoracic Surgery, Department of Surgery, Brown University/Rhode Island Hospital, Providence, RI, United States

**Keywords:** cardiac surgery, cardiopulmonary bypass, inflammation, ischemiareperfusion injury, organ damage

## Abstract

Cardiopulmonary bypass (CPB) initiates an intense inflammatory response due to various factors: conversion from pulsatile to laminar flow, cold cardioplegia, surgical trauma, endotoxemia, ischemia-reperfusion injury, oxidative stress, hypothermia, and contact activation of cells by the extracorporeal circuit. Redundant and overlapping inflammatory cascades amplify the initial response to produce a systemic inflammatory response, heightened by coincident activation of coagulation and fibrinolytic pathways. When unchecked, this inflammatory response can become maladaptive and lead to serious postoperative complications. Concerted research efforts have been made to identify technical refinements and pharmacologic interventions that appropriately attenuate the inflammatory response and ultimately translate to improved clinical outcomes. Surface modification of the extracorporeal circuit to increase biocompatibility, miniaturized circuits with sheer resistance, filtration techniques, and minimally invasive approaches have improved clinical outcomes in specific populations. Pharmacologic adjuncts, including aprotinin, steroids, monoclonal antibodies, and free radical scavengers, show real promise. A multimodal approach incorporating technical, circuit-specific, and pharmacologic strategies will likely yield maximal clinical benefit.

## 1 Introduction

The advent of cardiopulmonary bypass (CPB) revolutionized cardiac surgery and dramatically improved patient outcomes. CPB activates various inflammation, coagulation, fibrinolysis, apoptosis, and oxidation pathways. Exposure to nonphysiologic conditions during CPB leads to an intense immunologic response that can affect the function and recovery of multiple organ systems (1).

The body's immunologic response is designed to sequester and destroy what it recognizes as foreign. The initial stimulus or signal undergoes amplification due to redundant and synergistic inflammatory cascades. Frequently, this results in activation of both humoral and cellular components of the immune system, and the initial inflammatory response to CPB is no different. The activation of these pathways leads to release of multiple humoral mediators. Leukocytes, chiefly neutrophils, are then drawn to the site of production of these mediators, become activated, and subsequently adhere to endothelial cells by way of receptor interactions with adhesion molecules. Endothelial cells in turn become activated, rendering regional and remote capillary endothelium permeable to further migration of neutrophils and other intravascular molecules. Extravasation of neutrophils en masse results in release of large amounts of cytokines, chemokines, vasoactive substances, proteases of the coagulation and fibrinolysis systems, cytotoxins, and reactive oxygen species (ROS). Elimination of foreign antigen is the final step of this cascade of events.

Immunologic activation leading to inflammation is usually physiologic and protective, often leading to self-limited or subclinical organ dysfunction. However, on occasion, nonimmunologic activation that persists following CPB can be maladaptive and may progress beyond restoring homeostasis to marked fluid shifts and formation of microemboli, particularly in high-risk patients with limited functional reserve. This exuberant systemic inflammatory response to CPB, often characterized as systemic inflammatory response syndrome (SIRS), may manifest as clinically significant increase in capillary permeability, interstitial edema, and organ dysfunction. The link between CPB-induced inflammatory response and adverse clinical outcomes is still not well delineated. Several hypotheses have been proposed. One hypothesis suggests the balance between pro-inflammatory and anti-inflammatory cytokine release correlates with the magnitude of multiorgan injury (2). Temporal and magnitude changes in cytokine production patterns may further influence the clinical presentation and course of SIRS postoperatively (3, 4). A second hypothesis suggests SIRS is the result of a multifaceted response with overall cytokine upregulation leading to both a proinflammatory state (SIRS) as well as homeostatic, compensatory anti-inflammatory response syndrome (CARS) leading to systemic deactivation of the immune response, predisposing the patient to immunosuppression and infectious complications (5). The multi-hit hypothesis suggests CPB primes polymorphonuclear leukocytes such that subsequent exposure to stimuli that otherwise may be self-limiting (i.e., postoperative infection or ongoing ischemia) results in enhanced cytotoxin release and downstream organ dysfunction (5). This priming is thought to occur through various processes, including the secretion of cytokines, leading to pulmonary leucosequestration (5).

These harmful inflammatory effects are due to the interactions of a wide spectrum of compounds, including inflammatory triggers (complement-derived factors), mediators (cytokines and adhesion molecules), or effectors (proteolytic enzymes, oxygen free radicals, arachidonic acid metabolites, and immune cells). Hallmarks of ensuing multiorgan dysfunction include coagulopathy, myocardial dysfunction, pulmonary and renal insufficiency, neurocognitive deficits, hepatic injury, splanchnic bed hypoperfusion and bacterial translocation. Collectively, these multiorgan effects have been linked to post-perfusion/post-pump syndrome and ischemia-reperfusion injury.

Despite drawbacks attributed to the attendant inflammatory response, CPB remains a mainstay technique in cardiothoracic surgery, as it allows for adequate exposure of the lateral and posterior coronary arteries and facilitates a bloodless field in which to operate. While more drastic strategies have been employed to blunt excessive inflammatory response and its sequelae (including off-pump coronary artery bypass and minimally invasive techniques), an integrated approach to modulate the stress response incorporating pharmacologic measures and technical refinements has shown promise. Herein, we characterize the pathophysiology of the inflammatory response, and discuss potential strategies to intercept and attenuate this response.

## 2 Pathophysiology of inflammatory response following cardiopulmonary bypass

**Figure 1:**
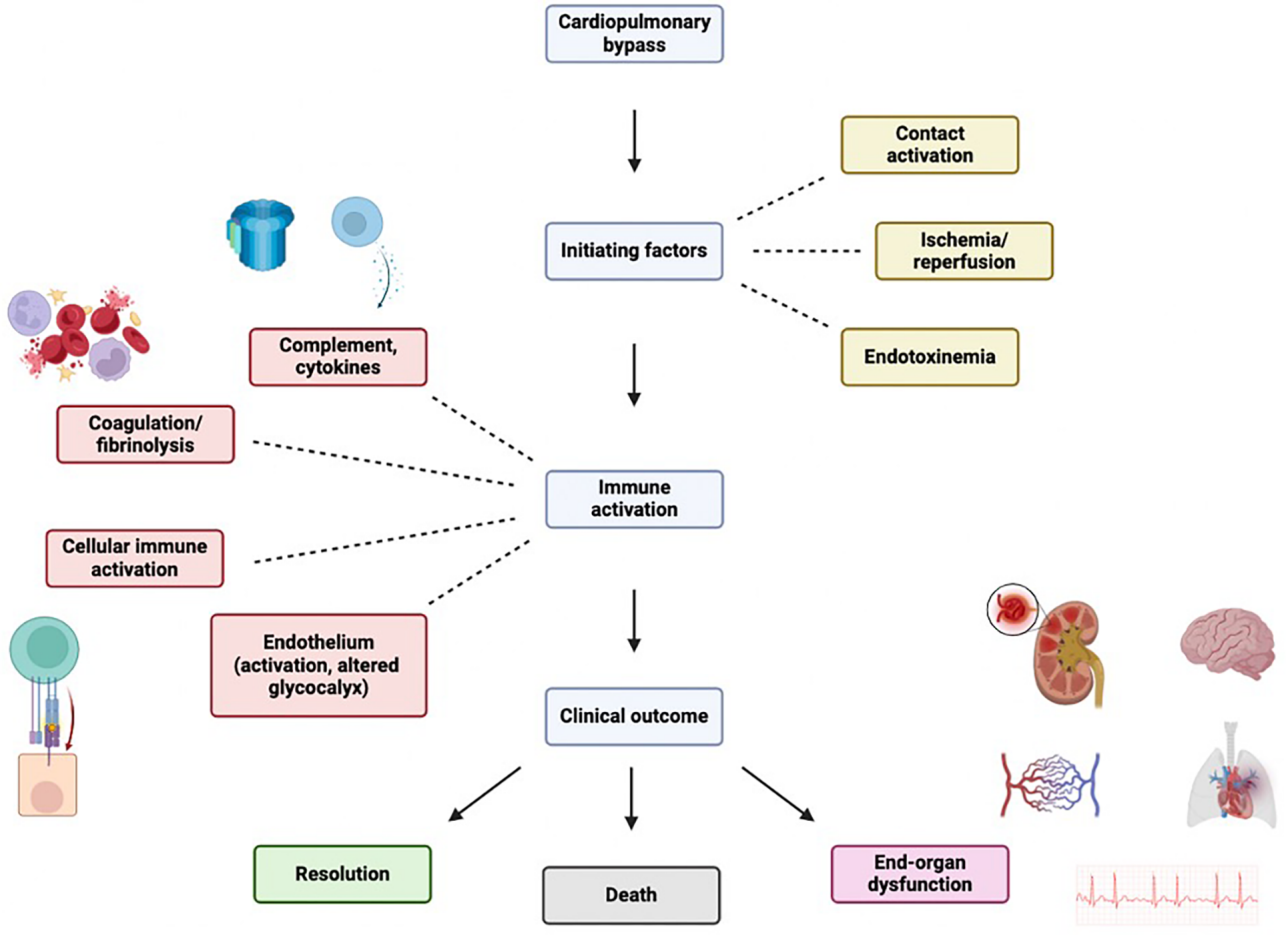
Inflammantory cascade following cardiopulmary bypass initiation. Figure created using Biorender.com.

Compared with off-pump approaches, CPB may trigger an intense physiologic response due to several stimuli (Figure 1) (6, 7):

• Blood contact with synthetic surfaces within the perfusion circuit and multiple tissues within the wound
• Abnormal blood-gas interface
• Pulsatile flow converted to laminar flow
• Hypothermia
• Surgical trauma
• Global myocardial ischemia during cardioplegic protection
• Ischemia-reperfusion injury to end-organs
• Endotoxemia proceeding from splanchnic hypoperfusion and bacterial translocation

The inflammatory response following CPB is multifactorial, and may become generalized and uncontrolled, leading to SIRS. The “early” phase is initiated by blood contact with nonendothelial surfaces of the extracorporeal circuit and ultimately involves both humoral and cellular constituents of the immune system. The “late” phase that perpetuates inflammatory cascades is characterized by ischemia-reperfusion injury, endotoxemia, coagulopathy, and heparin-protamine complex reactions (Table 1). The link between inflammatory, coagulation, and fibrinolytic cascades is complex and may partially be explained by acute phase reactions during CPB similar to those seen in sepsis (8). Another link may be nuclear factor kappa B (NFκB), a ubiquitous and inducible transcription factor that is implicated during all phases of the response but plays a central role in regulating pro-inflammatory genes during the acute phase reaction (9).

**Table 1:** Humoral and cellular factors influencing systemic inflammatory response associated with CPB.

Humoral	Cellular
Contact activation	Endothelial cell (EC) activation
Intrinsic coagulation	Adhesion molecules
Extrinsic coagulation	Leukocyte activation
Complement activation	○ Neutrophils
Fibrinolysis activation	○ Lymphocytes
Cytokines	○ Platelets
Endotoxin	○ Monocytes

### 2.1 Contact activation

**Figure 2:**
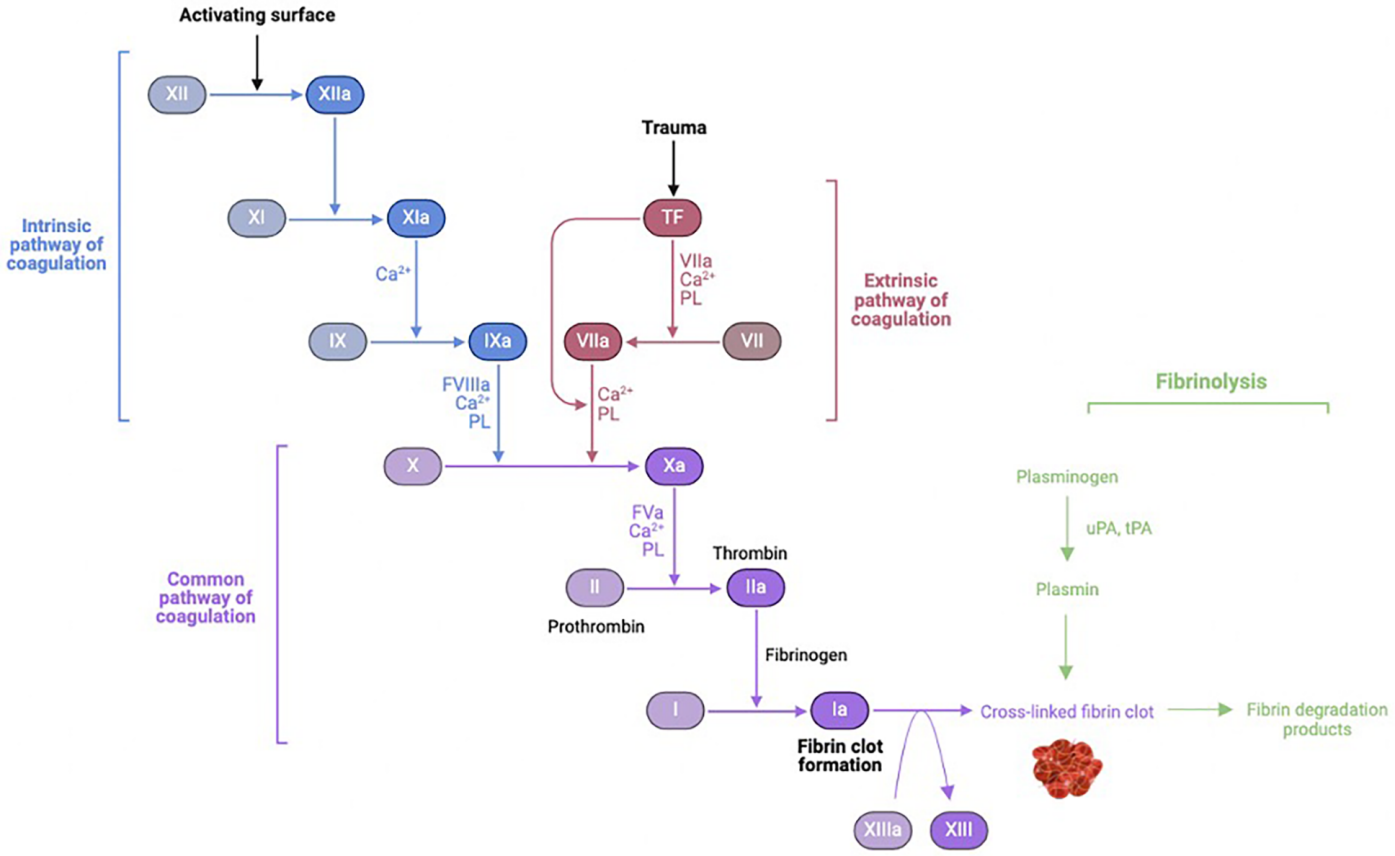
Coagulation-fibrinolysis pathway activation. uPA, urokinase; tPa, tissue plasminogen activator. Figure created using Biorender.com.

The exposure of blood to air and nonphysiologic surfaces of the extracorporeal circuit leads to simultaneous activation of coagulation and fibrinolysis cascades as well as the complement pathways of innate immunity (Figure 2).

Four proteins are involved in the contact activation pathway: factor XII (Hageman factor), factor XI, prekallikrein, and high-molecular-weight kininogen (HMWK). Upon exposure of blood to foreign material of the CPB circuit, factor XII is converted to its active form in the presence of prekallikrein and HMWK. Activated factor XII activates factor XI of the intrinsic pathway, ultimately leading to thrombin formation. Activated factor XII also converts prekallikrein to kallikrein. Kallikrein then: (1) feeds back to facilitate continued activation of factor XII, (2) induces cleavage of HMWK to form bradykinin, (3) potentiates mediators of the alternative complement pathway, and (4) promotes conversion of plasminogen to plasmin. Plasmin is an important link to the fibrinolytic cascade. Plasminogen is activated to plasmin by activated factor XII in the intrinsic pathway during contact activation, and by tPA as part of the extrinsic pathway later during CPB. In addition to thrombin, inflammatory mediators (cytokines and endotoxin) can activate plasminogen. Activated factor XII and plasmin both play a role in stimulating the classical complement pathway (10).

Activation of prothrombin to thrombin by way of the extrinsic pathway contributes significantly to systemic thrombin generation and thrombus formation. Circulating levels of tissue factor and activated factor VII increase following CPB and surgical trauma, correlating with stimulation of pro-inflammatory mediators interleukin-1 (IL-1), tumor necrosis factor-α (TNF-α), and endotoxin (11–13). CPB necessitates anticoagulation in the form of high-dose heparin to prevent immediate clotting within the circuit. Heparin potentiates antithrombin III to inhibit thrombin and other coagulation factors (activated X, IX, XI, XII, and kallikrein). During CPB, fibrinogen and fibrin are readily deposited onto circuitry, thus creating a surface to which thrombin avidly adheres. Upon binding to deposited fibrinogen/fibrin, thrombin undergoes a conformational change that renders it resistant to inhibition by heparin-activated antithrombin III. Thus, while heparin can inhibit systemic thrombin, it is unable to inhibit surface-bound thrombin. The surface-bound thrombin continues to generate more circulating thrombin, that can in turn activate a number of constituent blood elements, including platelets. Activated platelets can bind fibrin- and fibrinogen-coated surface of CPB circuitry, and also can individually provide a scaffold for prothrombinase complexes to form and convert prothrombin to thrombin. While high-dose heparin can limit fibrin-rich thrombus during CPB, it cannot prevent thrombin generation *per se*. Thrombin levels, measured as thrombin-antithrombin complex and prothrombin fragment, increase within minutes of initiating CPB, further increase following discontinuation of CPB, and persist for up to 60 days after surgery (14–17). Heparin concentrations, antithrombin III levels, and activated coagulation time are not associated with thrombin generation (10, 18).

Activated factor XII, thrombin, kallikrein, and products of fibrinolysis potentiate the inflammatory response. Activated factor XII and kallikrein stimulate neutrophil aggregation and degranulation. Thrombin induces endothelial cells to express receptors to facilitate neutrophil binding. Fibrinogen fragment D (D-dimer) disrupts the integrity of endothelial cells and stimulates continued complement activation.

### 2.2 Complement activation

Contact activation by biomaterials is a critical inciting event to downstream generation of humoral mediators through its activation of the alternative complement pathway. The CPB circuit lacks inhibitors normally found on endothelial cells that limit cofactor C3 binding and activation. This contact activation, potentiated by kallikrein, leads to activation of C3 and C5 (19). Their active split products, C3a and C5a, are anaphylatoxins that are potent chemoattractants (20). Their activity is mediated by complement receptor type 1 (CR1), a transmembrane glycoprotein expressed on leukocytes that regulates complement pro-inflammatory activity while inhibiting other complement pathways. C3a is a potent stimulator of platelet aggregation. There is a significant rise in plasma C3a levels with CPB associated with its duration (2), which is not observed when CPB is avoided (19, 21–26). C5a avidly binds to neutrophil receptors (19, 24, 27, 28) thereby stimulating them to be chemotactically drawn to sites of C5a production (29, 30), aggregate and adhere to endothelial cells (31–33), degranulate to release proteases (29, 34), and produce ROS (35). C5a levels are more difficult to directly measure as C5a is internalized rapidly upon binding to neutrophil receptors, but several studies have shown decreased available C5a receptors and increased levels of terminal complement complex C5b-C9, which provide evidence for C5a generation during CPB (28, 36).

Endotoxin is a powerful activator of the alternative complement pathway, with a smaller role in activating the classical complement pathway (25). Endotoxin, or lipopolysaccharide Lipid A, arises from cell walls of gram-negative bacteria and is released upon disruption of their cell walls. They may appear in circulation during CPB due to contamination of CPB circuitry, pulmonary arterial catheters, intravenous fluids, or banked blood production. The primary mechanism for endotoxemia, however, is due to splanchnic hypoperfusion and vasoconstriction during aortic cross-clamping and resultant transient gastrointestinal bacterial translocation (37–40). Transient ischemia and laminar flow in the gut increases intestinal permeability (37, 41), facilitating endotoxin release into circulation (42). Here, endotoxin can activate complement and stimulate production of inflammatory cytokines (43, 44). Endotoxin circulates in plasma by binding to LPS-binding protein. This complex binds to CD14 receptors on macrophages, enhancing TNF-α production (25, 45, 46). Endotoxin also stimulates endothelial cells to produce IL-6 (43). Levels of circulating endotoxin rise during and after CPB (25, 40, 42, 47–50), and are correlated with duration of aortic cross-clamping during CPB (48). Elevated LPS levels lead to myocardial dysfunction (46). Increased endotoxin levels in children with congenital heart defects undergoing CPB are associated with increased mortality after CPB (51).

CPB induces the classical complement pathway through contact activation (activated factor XII and plasmin) and heparin reversal with protamine, as evidenced by increased levels of the terminal complex C5b-C9, C3a, and C4a (27, 28, 52, 53). This further augments levels of C3a and C5a. The extent of complement activation has been correlated with duration of CPB (19). It remains unclear whether complement activation portends worse outcomes. While higher levels of C3a have been reported in those requiring prolonged mechanical ventilation, other groups have failed to identify a correlation between complement activation and acute lung injury or adverse hemodynamic responses (54–56). Equivocal findings may be due to the difficulty of parsing out the role of the complement system in the context of the complex inflammatory response associated with CPB (57).

### 2.3 Conversion to laminar flow

Following initiation of CPB, physiologic pulsatile aortic flow is converted to continuous laminar flow. Endothelial cells sense mechanical stresses through their attachments to the basement membrane and through membrane proteins on their luminal surface. Changes in mechanical stresses result in changes in downstream transcription of genes regulated by promoter regions containing shear stress-responsive elements. Conversion from pulsatile to laminar flow may induce expression of genes related to a pro-inflammatory phenotype. One group showed differential gene expression was responsible for a quiescent endothelial phenotype (lung Kruppel-like factor) after endothelial cells were exposed to pulsatile or laminar flow (58). Antioxidant proteins, including thioredoxin reductase and ferritin, were among shear-regulated gene products. Other studies have shown Mn^2+^- and Cu^2+^/Zn^2+^-superoxide dismutase to be shear-regulated as well (59). Reduced activation of transcription factor NFκB and expression of pro-inflammatory cytokines, including IL-6, TNF-α, and IL-1 (60, 61) as well as decreased endothelial activation (62, 63) in the group undergoing pulsatile perfusion compared to the group undergoing non-pulsatile perfusion has been described.

### 2.4 Oxidative stress

Free radicals are molecules with unpaired electrons that render them highly reactive. ROS are free radicals derived from oxygen, and can be formed by activated neutrophils during their cytotoxic oxidative burst as a response to C5a stimulation (64–66). Exposure of blood to material of the CPB circuit as well as ischemia-reperfusion both generate various ROS implicated in oxidative damage, including superoxide anion, hydrogen peroxide, hydroxyl radical, peroxynitrite, hypochlorous acid, and singlet oxygen. Natural defense mechanisms to neutralize ROS and restore balance (redox state) include enzymes and free radical scavengers. Antioxidant enzymes include superoxide dismutase, glutathione peroxidase, and catalases. Mitochondrial scavenger complexes integrate thioredoxin and peroxiredoxin proteins (67–70). Should the body's detoxifying capacity be outstripped, ROS can cause direct damage to endothelial cell and fibroblast membrane lipids compromising membrane integrity, render proteins dysfunctional, induce nucleic acid damage that results in downstream changes in transcriptional programs via NFκB modulation, and provide positive feedback to inflammatory cascades.

Direct measurement of ROS is difficult given its short half-life and highly reactive properties, but indirect methods via measurement of more stable intermediates have shown increased ROS activity during and after CPB (24, 71–74). The onset of CPB and aortic cross-clamping creates transient ischemia, subjecting the myocardium and other organs to direct hypoxic cellular damage. CPB itself may induce generation of ROS in the area drained by the inferior vena cava (73, 74) as well as systemic oxidative stress (75). Reperfusion occurs upon removing the aortic cross-clamp, which generates further oxidative stress through recruitment of activated neutrophils to post-ischemic tissue. Following reperfusion, ROS may impair nitric oxide (NO) availability and predispose myocardial vessels to spasm and thrombose (76–78). A correlation has also been observed between timing of lipid peroxidation and degree of complement activation (24). Hypothermia may also influence ROS production by altering neutrophil-endothelium interactions during CPB (79–82).

### 2.5 Cytokines

Cytokines are another major group of humoral mediators that play a central role in inflammation and cell signaling. The body produces cytokines constitutively whereby subsets of immune cells maintain baseline levels of cytokines under normal conditions. Cytokines must bind cell membrane receptors to exert their effects, and the action of one or more cytokines is necessary to mount an immune response. These molecules form an intricate network in the development of inflammation, as the production of one cytokine influences the synthesis or response of others. The overlapping actions of different cytokines can be explained by both pleiotropy (single cytokine: multiple effects) and redundancy (multiple cytokines: same effect). The ability of certain cytokines to signal via more than one type of receptor complex also contributes to their pleiotropic actions wherein separately activated pathways contribute to distinct downstream effects. Simultaneously, redundant actions of cytokines allow for signal amplification; different cytokine receptors with similar motifs mediate coupling to other processes, ultimately leading to activation of converging inflammatory pathways in potentiating the immune response.

Broadly, cytokines include tumor necrosis factor, interleukins, interferons, and several growth factors. The production and release of cytokines is induced by complement factors and their degradation products during CPB-related acute phase reaction. A number of other factors may also contribute, including endotoxin, oxidative stress, ischemia-reperfusion, and effects of cytokines themselves. While cytokines are generally considered to be products of mature leukocytes, their secretion may be modulated by other cell types, including platelets and endothelial cells (83–90). The degree of the inflammatory response is strongly influenced by the balance between pro-inflammatory (TNF-α, IL-1, IL-6, and IL-8) and anti-inflammatory (IL-10) cytokines (3).

#### 2.5.1 Tumor necrosis factor α (TNF-α)

Tumor necrosis factor α (TNF-α, or cachectin) functions within a complex and tightly regulated cytokine network. It has a role in orchestrating the inflammatory response by inducing expression of other pro-inflammatory cytokines (IL-1 and IL-6) as well as increasing its own production. It functions in cell signaling through interactions with p55 and p75 receptors localized to the myocardium (91). It induces nitric oxide synthase and therefore increases concentrations of nitric oxide following CPB. It usually peaks shortly after surgery and then undergoes rapid degradation, but excessive production may lead to organ dysfunction. In the lung, it induces apoptosis and has been implicated in pulmonary complications. Infusion of antibodies to TNF-α prevented pulmonary edema, improved oxygenation, and significantly reduced markers of inflammation (neutrophil count, plasma TNF-α levels, malondialdehyde concentrations) in a rabbit model (92). TNF-α has been implicated in renal dysfunction as it induces fibrin deposition in the kidney glomerulus, promoting cell infiltration and vasoconstriction, thereby reducing glomerular filtration rate (93). TNF-α and IL-1 synergistically suppress myocardial contractility through a mechanism mediated by sphingosine impeding calcium-induced calcium release from the sarcoplasmic reticulum. This may result in low cardiac output, decreased vascular smooth muscle tone, and development of thrombosis, thereby contributing to dysfunction and hemodynamic instability after CPB (46, 94–96). Trends in TNF-α levels following CPB are variable, as some studies have shown increased levels while others have failed to demonstrate this. Additionally, inter-individual differences in TNF-α production may be attributed to genetic variability (97). In contrast to other cytokines (i.e., IL-6), there is no evidence that indicates TNF-α is released in large amounts following CPB (98).

#### 2.5.2 Interleukins

Interleukins (IL) comprise a broad group of cytokines that function as intermediaries between different leukocytes and regulate various stages of the inflammatory response. IL-1 is an endogenous pyrogen. Levels of IL-1β usually increase after CPB, but this cytokine is difficult to detect due to hemodilutional effects of CPB. The IL-1 response pattern is consistent with its role as a key mediator of inflammation, both through its synergistic actions on TNF-α as well as its induction of other pro-inflammatory cytokines, including IL-6. IL-6 is a pleiotropic cytokine role that chiefly coordinates the acute phase reaction. It also induces the expression of adhesion molecules on cardiac myocytes to facilitate neutrophil adhesion, inhibits apoptosis in various cell types, enhances antibody production by activated B lymphocytes, and has negative inotropic effects on cardiac myocytes through induction of local nitric oxide release (99–104). In this way, it may be more a precise marker for progression of inflammation after CPB. A marked increased in IL-6 occurs during CPB, peaking within a few hours following CPB (105, 106), with a gradual decrease toward preoperative levels within 24 h (28). Peak IL-6 concentrations were a function of aortic cross-clamping duration. This characteristic trend in IL-6 levels has been observed in the setting of bubble and membrane oxygenators (105), after hypothermic and normothermic CPB (107), and with and without heparin-coating CPB circuitry (28). The magnitude of increase in IL-6 levels was positively correlated with duration of CPB but not duration of aortic cross-clamp time (108). In the pediatric population, duration of CPB and aortic cross-clamp time were attributed to pronounced postoperative inflammation, with only a modest influence of the degree of hypothermia (109). Rise in IL-6 after CPB has been correlated with increasing age, as those older than seventy years had a greater increase in plasma IL-6 levels during ischemia and reperfusion than their younger counterparts (110). Postoperative IL-6 levels are significantly higher in patients with complications compared to those without (111). Increased levels of IL-6 have been associated with myocardial dysfunction and wall motion abnormalities (112, 113), while effects on hemodynamics are less clear (114). Rise in IL-6 has not been correlated with complement activation.

IL-8 is a potent chemotactic agent involved in the homing of neutrophils and macrophages sites of inflammation (115). It may also play a role in ischemia-reperfusion injury, as postoperative cardiac troponin-I levels correlate strongly with IL-8 levels in patients following coronary artery bypass grafting (CABG) (116, 117). It has been implicated in precipitating vascular damage, particularly in the lungs and kidneys. Increased levels of IL-6 and IL-8 following CPB have been reported (81, 106, 107, 113, 118–123), and correlate with duration of cardiac ischemia during CPB and regional wall motion abnormalities (113, 118).

IL-10 has anti-inflammatory properties via its downregulation of pro-inflammatory cytokine synthesis by type 1 T helper cells, neutrophils, and monocytes (124–126). It has been associated with decreased production of TNF-α, IL-1, IL-2, and interferon-γ, ROS and nitric oxide derivatives (124, 127, 128). While several pro-inflammatory cytokines (TNF-α, IL-6, and IL-8) have been shown to originate from myocardium (120, 121, 129), the liver has been shown to be the primary source of IL-10 in patients undergoing CPB (120, 121, 130, 131). Rapid and transient secretion of IL-10 has been noted following CPB (132, 133). *In vivo* kinetics of IL-10 release are similar to those observed in murine endotoxemia experiments following lipopolysaccharide challenge but contrast with *in vitro* data from human monocytes following lipopolysaccharide stimulation (134, 135). IL-10 produced during CPB may represent an *in vivo* regulatory mechanism for controlling activation of cells that synthesize pro-inflammatory cytokines.

There are several caveats in relating serum cytokine levels associated with CPB to organ dysfunction. Plasma concentrations often do not reflect local effects. Several cytokines may also avidly bind to other plasma proteins, leading to inaccurate detection. Inter-individual genomic differences also contribute to heterogeneity in cytokine levels, and have implications for identifying therapeutic targets and developing preventative strategies that can be broadly applied. Genetic variants of promoter regions encoding IL-6 (−174 G/C polymorphism, −572 G/C polymorphism), TNF-α (308 G/A polymorphism), and antioxidant response elements (NQO1) have been linked to increased cytokine production and postoperative complications (136–142). A prevalent single-nucleotide polymorphism (SNP) of the gene encoding IL-18 was shown to be associated with increased TNF-α levels and decreased IL-10 levels. Apolipoprotein E4 allele has been correlated with increased IL-8 and TNF-α generation and decreased IL-10 levels, with a speculated link to postoperative cerebral injury (143–145). While the A allele of this SNP was associated with 30-day and 50-year mortality in the INFLACOR study, post-hoc analysis revealed the C allele of −572 G/C polymorphism to be significantly associated with reduced benefit of prophylactic administration of dexamethasone on postoperative IL-6 levels compared to the G allele. Its effects on C-reactive protein (CRP), however, did not appear to be genotype-dependent. These genomic differences pose a challenge to randomized controlled trials (RCTs) evaluating clinically relevant responses to prophylactic measures. Second-generation studies, including genome-wide association studies (GWAS), may be able to more completely evaluate genotype-phenotype relationships.

### 2.6 Cellular immune activation

CPB-induced cellular immune activation plays a key role in the ensuing inflammatory response (146). Recruitment of immune cells is mediated by upregulation of cytokines, chemokines, complement system proteins, and adhesion molecules, including selectins and integrins (147). Primary adhesion occurs when freely moving neutrophils are converted to the “rolling” state following upregulation of P- and E-selectin on endothelium and upregulation of L-selectin on neutrophils. This leads to neutrophils initially traveling in the center of postcapillary venules to tumble along endothelial walls endothelium-neutrophil selectin interactions. C5a, which is released in response to contact activation during CPB, is a potent stimulator of endothelial P-selectin expression (148). E-selectin subsequently replaces P-selectin on endothelium to maintain primary adhesion. Secondary adhesion of neutrophils is mediated by integrins. Integrins CD11a/CD18 and CD11b/CD18 are expressed by neutrophils; IL-8 and C5a are potent stimulators of CD11b/CD18 expression on neutrophils (147, 149, 150). Activated integrins bind adhesion molecules on endothelium (intercellular adhesion molecule-1 and intercellular adhesion molecule-2) and extracellular matrix elements including fibrinogen (147). Both selectins and integrins have been shown to increase following CPB (150). Transmigration of neutrophils follows secondary adhesion (151). CD11b/CD18 binding to endothelium during secondary adhesion and transmigration primes neutrophils to degranulate and undergo respiratory burst for up to 24 h following CPB (49, 147, 151). Elastase, myeloperoxidase, and ROS released from neutrophils result in cytotoxic damage to endothelium and tissues (49, 66, 151). Modulation of adhesion, transmigration, and neutrophil priming is heavily influenced by platelet-activating factor, IL-8, and C5a (147, 152). Normalization of C5a levels limits the extent of CPB-induced inflammatory response (149). Increased secretion of monocyte chemoattractants leads to upregulation of selectins and integrins and further activation of circulating monocytes and macrophages. Naïve monocytes and granulocytes are hyperstimulated following exposure to post-CPB plasma. Impaired oxidative burst and phagocytic activity have been observed 48 h following CPB, suggesting a biphasic course characterized by early tissue cytotoxicity and late neutrophil dysfunction (146, 153, 154).

Clinically, increased cellular immune activation leads to pulmonary leukocyte sequestration that has been associated with severe histologic lung injury (155, 156). Inhibition of CD11/CD18 expression or function improves myocardial function following CPB, and neutrophil adhesion blockade reduces pulmonary injury during CPB (157–159). Reduction of circulating activated leukocytes has been associated with reduced organ injury (160).

### 2.7 The endothelium and glycocalyx degradation

The endothelium is central to inflammatory pathophysiology following CPB (161). The endothelial glycocalyx protects the endothelial cell monolayer and is composed primarily of transmembrane heparan sulfate and syndecan proteoglycans. The glycocalyx plays important roles in leukocyte and platelet adhesion following immune activation, vascular permeability facilitating leukocyte transmigration, and regulation of the coagulation cascade on the luminal endothelial surface (161–163). Following CPB, activated metalloproteinases and TNF-α induce shedding of syndecan-1 and heparin sulfate from the glycocalyx; increased plasma levels of these glycocalyx breakdown products following CPB initiation have been extensively documented (164–170). Membrane-bound syndecan-1 reduces cytokine production while soluble syndecan-1 results in neutrophil activation and monocyte chemotaxis (171–173). Pro-inflammatory IL-6 and IL-8 levels at two timepoints (preoperatively and 6 horus postoperatively) and anti-inflammatory IL-10 have been correlated with CPB-induced inflammation (174). Syndecan-1 levels have been shown to prognosticate acute kidney injury following CPB in children though levels vary widely between patients and do not correlate intraoperatively (174, 175).

## 3 Strategies to modulate CPB-associated inflammatory response

**Figure 3:**
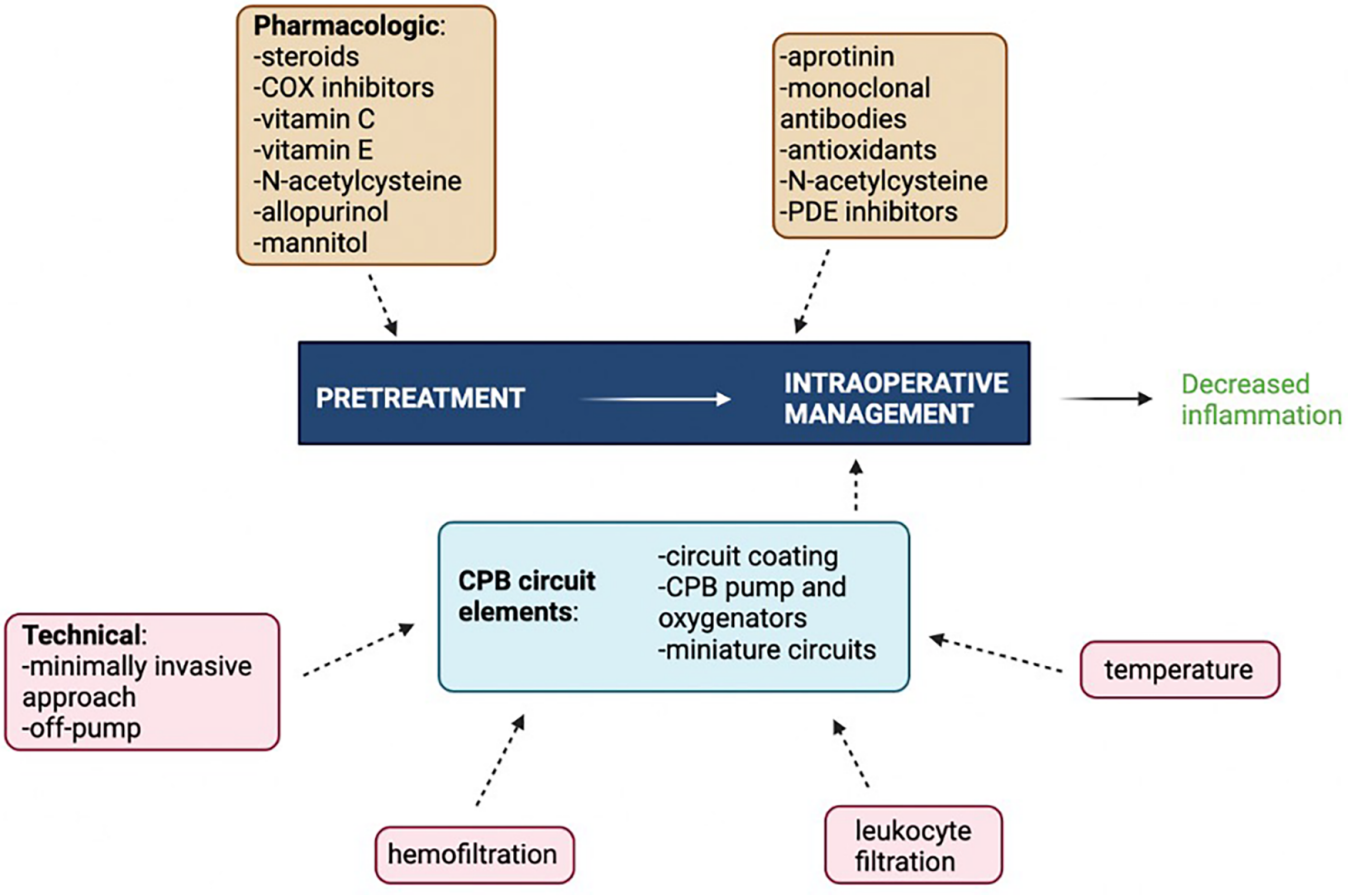
Multimodal approach to attenuating CPB-associated inflammation. COX, cyclooxygenase; PDE, phosphodiesterase. Figure created using Biorender.com.

Innovations and potential therapeutic targets have been investigated to better modulate the inflammatory response to CPB. The main challenge remains to balance mitigating the effects of excessive inflammation and immune activation while still preserving host defenses and wound healing (176). The most clinically effective way to curb CPB-associated inflammation involves targeting multiple inflammatory mediators simultaneously using a combination of surgical, pharmacologic, and mechanical pump approaches as no single intervention is supported by strong level A evidence (Figure 3). Interventions with level A evidence include off-pump surgery, miniaturized CPB circuits, coated circuits to improve biocompatibility, leukocyte filtration, complement (C5) inhibition, preoperative aspirin, and corticosteroid prophylaxis. Interventions with level B evidence include, but not limited to, hemofiltration, aprotinin, nitric oxide donors, C1 esterase inhibition, neutrophil elastase inhibition, N-acetylcysteine, and intensive insulin therapy (177). The precise combinations of studied interventions tailored to specific patient populations have yet to be determined.

### 3.1 Non-pharmacologic strategies

#### 3.1.1 Coated CPB circuits

Heparin-coated circuits improve biocompatibility of the extracorporeal circuit, and translation into clinical benefit has been demonstrated in certain populations (178, 179). Heparin molecules bound to the surface of the CPB circuit resemble heparin sulphate glycosaminoglycans on endothelial cells and reduce direct contact of blood with the otherwise artificial, nonendothelial material lining the CPB circuit. These coated circuits improve biocompatibility through various mechanisms. Heparin-coated circuits reduce cytokine release, complement activation *in vivo* and *in vitro*, kallikrein, and leukocyte activation (180–184). They have been shown to reduce platelet adhesion and improve platelet function, as well as inhibit the release of pro-inflammatory cytokines TNF-α, IL-6, IL-8 as well as soluble TNF receptors (180, 185–187). Significant reduction in mean concentrations of polymorphonuclear (PMN) elastase and soluble C5b-9 (*p* < 0.001 and *p* = 0.006, respectively) at one hour following initiation of CPB have been reported. Heparin coating also reduced postoperative blood loss (188–190). An RCT conducted in the Netherlands showed reduced complement activation that correlated with improved clinical performance scores in patients who underwent CPB with heparin-coated circuits in combination with full systemic heparinization (191). A large, multicenter RCT demonstrated improved clinical outcomes, shorter length of stay (hospital and ICU), and decreased respiratory and renal dysfunction in high-risk patients (192). A meta-analysis of 41 RCTs showed heparin coating was associated with 40% reduction in re-sternotomy rates (*p* = 0.002) and 20% reduction in patients requiring blood transfusion but no significant difference in 24-h blood loss or adverse events. Heparin coating reduced average ventilation time by 80 min, ICU length of stay by 9 h (*p* < 0.001), and average hospital length of stay by 0.5 days (*p* = 0.02) (193). In cardiac reoperations, use of heparin-coated circuits with full systemic heparinization was associated with decreased rates of reoperation for bleeding (*p* = 0.058) and lower blood transfusion requirement (*p* = 0.035) without significant difference in adverse events (189).

A study investigating heparin-coated circuits with administration of reduced systemic heparin dose showed decreased intraoperative platelet counts, maximal intraoperation concentrations of platelet factor 4 and PMN elastase, as well as 12-h blood loss with heparin-coated circuits but no difference adverse events or 30-day mortality (190). A departmental analysis revealed patients undergoing CPB with heparin-coated circuits received lower dose of systemic heparin, showed improvements in clinical parameters, and had a decreased rate of adverse events (10%, *p* = 0.035) compared to their counterparts (194).

Heparin-albumin and polymer-coated circuits as well as phosphorylcholine-coated circuits have been shown to induce fewer inflammatory responses and oxidative stress compared to other circuits (195, 196). However, heparin-coated circuits are associated with better preservation of endothelial glycocalyx compared with phosphorylcholine-coated circuits (197). An RCT investigating albumin-coated circuits in patients undergoing aortic arch replacement with deep hypothermic circulatory arrest showed mitigation in platelet reduction as evidenced by decreased transfusion requirement but no effect on platelet dysfunction (198). A study investigating hyaluronan-based heparin-coated circuits in various risk cohorts showed improved platelet preservation and better perioperative outcomes; ventilation time, hemorrhage, and degree of inflammation were reduced in high-risk groups, which translated to shorter ICU and hospital length of stay (*p* = 0.001 and *p* = 0.006, respectively) (199). New data is still emerging for pediatric populations. A randomized pilot study recently demonstrated application of new ternary polymer, SEC-1 coat™ in pediatric cardiac operations improved biocompatibility with regard to platelet preservation and attenuated coagulation activation and overall inflammatory reaction (200). Another study showed no purported benefit in improving coagulation derangements during pediatric CPB as assessed by primary endpoint of concentration of β-thromboglobulin across all time points, as well as secondary endpoints of other markers of coagulation and platelet function (201).

Multiple studies found reductions in inflammatory markers, marginal improvement in biocompatibility, but minimal or no correlation with improved outcomes (202, 203). One such study showed reduced complement activation and synthesis of pro-inflammatory cytokines but no significant differences in fibrinolysis, platelet activation, time to hemostasis, postoperative blood loss at 12 h, total blood transfusion requirement, or intubation time (202). Another multicenter trial similarly found decreased complement activation but no association with release of specific neutrophil granule enzymes, myeloperoxidase, lactoferrin, or clinical outcome (203). A multimodal approach incorporating heparin-coated circuits, high-dose aprotinin, and pre-CPB hemofiltration reduced inflammatory response and improved clinical outcomes in high-risk patients (179).

Overall, heparin-coated circuits and newer third-generation heparin-polymer-coated circuits induce fewer inflammatory responses and are associated with improved outcomes and, therefore, justify their additional cost. Newer third-generation circuits preserve platelet function and improve perioperative outcomes, including reduced blood loss, reoperation rates, ventilation, time, and length of stay.

#### 3.1.2 Hemofiltration

Hemofiltration, or ultrafiltration, removes excess fluid and low-molecular-weight substances from plasma through a hydrostatic pressure gradient. It has been shown to improve hemodynamic parameters, cardiac and pulmonary function, and reduce inflammation with greatest benefit in pediatric patients. It has been associated with reduced levels of TNF-α, IL-1, IL-6, IL-8, C3a, and myeloperoxidase levels postoperatively in this population (204–208). Clinical benefits include improved hemodynamic stability and early postoperative oxygenation and decreased postoperative blood loss and duration of mechanical ventilation. It has been associated with decreased endothelin-1, potentially explaining improvement in pulmonary hypertension following congenital heart surgery (204, 206, 207, 209–211). Modified ultrafiltration has been associated with improved left ventricular systolic function and diastolic compliance, increased blood pressure, and reduced inotropic drug administration in the early postoperative period in infants (212–214). Hemofiltration has shown less clinical advantage in adults as it is less effective in removing pro-inflammatory cytokines (215). However, modified hemofiltration has been associated with decreased early morbidity, postoperative bleeding, and blood transfusion requirements in adults and its use was not associated with hemodynamic instability in adults (215–217).

#### 3.1.3 Leukocyte filtration

Activated leukocytes form the first line of defense against foreign substances and are critical players in potentiating the inflammatory response. Leukocyte-specific filters that trap activated neutrophils and monocytes implicated in SIRS attenuate inflammation and oxidative stress and have been associated with improved outcomes in the immediate postoperative period (218–222). Filters decrease concentrations of circulating platelets and leukocytes by interfering with endothelial-mediated leukocyte activation and subsequent neutrophil transmigration. This effectively decreases the endothelial-mediated component of the CPB-associated inflammatory response.

Leukocyte filtration may limit postoperative myocardial and pulmonary dysfunction following CPB. Results of a prospective randomized study showed patients undergoing coronary revascularization had decreased total leukocyte counts during and after CPB, and significantly decreased activated leukocyte counts at all timepoints. The rate of alveolar exhaled NO production and alveolar-arterial oxygenation index were significantly increased in the control group, where NO was a marker for lung inflammation. Leukocyte depletion was also associated with lower pro-inflammatory cytokine (IL-6 and IL-8) burst postoperatively in those with normal preoperative oxygenation capacity. There were no differences in intubation time, ICU, or hospital length of stay (223–226). Though, as expected, the rate of alveolar NO production increased in both groups following CPB, absolute NO production was shown to be lower with leukocyte depletion, suggesting filtration may be lung-protective. Leukocyte depletion at early reperfusion in those with limited preoperative oxygenation capacity (mild lung dysfunction and chronic obstructive pulmonary disease) and with increased duration of CPB time was associated with improved oxygenation, shorter intubation time, and shorter ICU and hospital length of stay (227, 228). Filtration may improve postoperative lung function by mitigating pulmonary reperfusion injury. Leukocyte depletion of residual blood prior to re-transfusion also improved lung function. In patients undergoing urgent CABG for unstable angina, leukocyte depletion of re-transfused blood and during CPB reduced markers of myocardial injury, whereas leukocyte depletion did not confer clinical benefit in low-risk patients (229). In patients with decreased left ventricular function, leukocyte depletion of blood cardioplegia alone improved early myocardial function and attenuated myocardial injury (160, 230). In those with left ventricular hypertrophy undergoing valve surgery, terminal blood cardioplegia reduced myocardial injury and improved heart function (231, 232). A large-scale clinical trial showed reduced overall 60-day mortality with leukocyte depletion of transfused blood, mainly attributed to reduction in noncardiac causes of death including multisystem organ failure. In those who received greater than 3 units of blood, postoperative infection rate was lower with leukocyte depletion (233). More recently, a study showed preoperative neutrophil response to *in vitro* stimuli may predict clinical outcome following CPB, but leukocyte filtration did not offer significant benefit (234).

Leukocyte depletion may provide renal protection. A prospective randomized study of in 40 patients undergoing CABG showed leukocyte filtration decreased indices of glomerular and tubular injury, namely microalbumin/creatinine ratio, urinary excretion of microalbumin, and retinol-binding protein (235). Filtration of neutrophils containing myeloperoxidase decreased apoptosis, caspase-3 activity, and IL-1β activation and effectively improved post-ischemic renal function and structure in a porcine model of isolated kidney perfusion (236).

#### 3.1.4 Cytokine adsorption

Extracorporeal blood purification through hemoadsorption utilizes biocompatible highly porous nonpolar polymer sorbent beads to sequester hydrophobic cytokines based on size exclusion and concentration-dependent surface adsorption throughout the beads. Nonspecific adsorptive characteristics allow for reduction in circulating pro-inflammatory cytokines, sequestration of free hemoglobin and bilirubin, and fortifying the endothelial glycocalyx. Clinical benefits include improved hemodynamic and metabolic stabilization postoperatively. No adverse effects of hemolysis or leukocyte removal have been reported. The first RCT investigating hemoadsorption with Cytosorb in cardiac surgical patients found prolonged anti-inflammatory IL-10 effect in the treatment group (237). A trial at a single center in Switzerland showed neither increased nor decreased cytokine levels (pro- or anti-inflammatory) with use of Cytosorb (238). Results showed no change in relevant clinical outcomes though the procedure was both feasible and safe. The REFRESH 1 pilot RCT showed Cytosorb signgicantly reduced C3a and C5a, as well as plasma-free hemoglobin during valve replacement operations (239). When comparing hemoadsorption to glucocorticoids, methylprednisolone was shown to more effectively reduce inflammatory markers (IL-6, TNF-alpha, and IL-8) though no differences in cardiac index or parameters of clinical outcomes were reported (240, 241). An RCT investigating intraoperative hemoadsorption showed clinical benefit as evidenced by reduced incidence of severe acute respiratory distress syndrome and a trend toward shorter ventilation times (242).

#### 3.1.5 Temperature

Studies comparing the acute phase reaction associated with normothermic vs. hypothermic CPB have conflicting results. This is due in part to inconsistent definitions of hypothermia. Hypothermia has been shown to delay but not completely prevent the expression of inflammatory mediators as increased levels of adhesion molecules and leukocyte proteolytic enzymes were seen at 34°C compared to moderate hypothermia (26°C–28°C) (80–82). Still other studies have shown no difference between patients randomly assigned to undergo CPB at various temperatures: > vs. 27-° in one study; 28°C, 32°C, vs. 37°C in another study (243, 244). Increased levels of NO were seen with CPB at 34°C when compared to CPB at 28°C, resulting in reduced systemic vascular resistance (245). A prospective randomized study in patients undergoing valve operations showed those undergoing normothermic CPB had similar levels of myocardial protection as measured by dynamics of troponin I while those undergoing hypothermic CPB benefitted from significantly lower ventilation times (*p* = 0.01) (246). A study investigating 262 different proteins using high-throughput technology showed deep hypothermic circulatory arrest (DHCA) and rewarming potentially exert a significant effect on the plasma proteome in patients undergoing aortic operations as evidenced by suppression of complement activation during hypothermia. These findings were confirmed by changes in terminal complement complex (C5b-9) levels. Following rewarming, however, these levels of terminal complement complex were more increased with DHCA than with normothermic CPB while 48 other proteins were significantly downregulated (247). In patients with left ventricular dysfunction, normothermia was found to enable decreased requirement for defibrillation after aortic unclamping and postoperative cardiac pacing, translating to improved myocardial protection. Normothermia had no effect on development of postoperative stroke, atrial fibrillation, renal failure, or mortality (248). A large-animal study recently showed hypothermic CPB attenuated platelet degranulation and coagulopathy and better maintained oxygenator performance in swine (249). Several RCTs studying pediatric populations showed normothermic CPB as noninferior to hypothermic CPB with endpoints including inotrope duration, intubation time, hospital stay, and early neurodevelopmental outcomes in low-risk populations (250, 251).

#### 3.1.6 CPB pumps and oxygenators

Studies evaluating the potential benefit of pulsatile pumps have not yielded conclusive results regarding clinical outcomes. Few studies have reported reduced levels of endotoxin and other pro-inflammatory mediators while other studies have not. Routine use of centrifugal pumps for CPB has not shown clear clinical advantages compared to roller pumps, and in fact several studies have showed increased levels of anaphylatoxins, pro-inflammatory cytokines, adhesion molecules, and leukocyte elastase with the use of centrifugal pumps. Radial-flow-patterned oxygenators may limit the extent of inflammation triggered by oxygenators in CPB when compared axial-flow-patterned oxygenators as evidenced by the results of a recent prospective RCT that noted significantly lower levels of humoral inflammatory markers (IL-1, IL-6, and TNF-α) 24 h postoperatively (252). In pediatric patients, controlled reoxygenation during CPB has shown benefit in single-ventricle patients as evidenced by reduced markers of organ damage, inflammation, and oxidative stress when compared to their double-ventricle counterparts (253).

#### 3.1.7 Miniature CPB circuits

Miniature extracorporeal circulation (MECC) systems have been developed to eliminate blood-foreign surface interface, shorten tubing length, reduce priming volume, eliminate venous reservoirs and cardiotomy suction, minimize hemolytic and consumptive effects on blood cells, and maximize blood re-transfusion (254). These systems consist of a centrifugal pump, membrane oxygenator, and arterial filter with all components of the system coated with heparin to optimize biocompatibility. An RCT confirmed a milder induced inflammatory response compared to conventional CPB with reduced levels of IL-6, TNF-α, and elastase release. A more recent RCT also showed reduced migration inhibitory factor levels associated with MECC systems in addition to decreased release of pro-inflammatory cytokines in the immediate postoperative period. This overall reduction correlated with decreased blood transfusion requirement and shorter mechanical ventilation time on bypass (255). The initially reduced levels of inflammatory markers seen with MECC may not be sustained throughout the postoperative period. An RCT investigating type 2 MECC compared to conventional CPB circuit in 50 patients undergoing aortic valve replacement found significantly lower levels of pro-inflammatory markers at 2 h postoperatively (*p* = 0.013) but no difference at 24 h (*p* = 0.990) when adjusting for type of oxygenator and hemoglobin. MECC was still associated with shorter perfusion times and less transfusion requirements (256). A small prospective RCT showed decreased IL-6, decreased hemolysis peaks as evidence by plasma-free hemoglobin levels, higher cardiac index and reduced pulmonary vascular resistance within 30 min postoperatively associated with MECC (257). However, these differences were not significant, and larger prospective RCTs are lacking. Normothermic CPB using MECC systems may be beneficial for perioperative preservation of pulmonary function and hemostasis in low-risk patients (258). These systems offer a promising minimally invasive approach to CPB.

#### 3.1.8 Minimally invasive and off-pump cardiac surgery

Advances in minimally invasive cardiothoracic surgery, including laparoscopic and thoracoscopic operations, allow for comparable outcomes while avoiding a full median sternotomy. The reduced size of surgical incisions significantly decreases the inflammatory response, but these approaches have not infrequently been associated with increased duration of CPB and aortic cross-clamping time. These results have been seen with minimally invasive valve operations as well as minimally invasive pulmonary embolectomy. While ventilator time and ICU length of stay were similar, minimally invasive operations resulted in decreased overall hospital length of stay by almost 5 days (259). In a retrospective analysis comparing video-assisted thoracoscopic surgery (VATS) vs. open operation for mitral valve disease, VATS was associate with longer duration of CPB and aortic cross-clamping but decreased ventilation time and ICU length of stay. Clearance of lactate was increased while levels of pro-inflammatory C-reactive protein, neutrophil-lymphocyte ratio, and cardiac troponin levels were significantly decreased at 24 h postoperatively in those undergoing VATS (260). These results suggest a totally thoracoscopic approach may be superior to conventional median sternotomy with regard to extent of inflammatory reaction, cardiac injury, and postoperative recovery (260).

Miniature aortic valve replacement (mini-AVR) has been evaluated in recent studies. A prospective RCT showed minimal difference in operative time on CPB, cross-clamp time, and overall operating time when evaluating patients undergoing AVR through median sternotomy compared to right anterolateral thoracotomy (261). However, cosmesis and patient satisfaction were significantly higher with reduced length of incision associated with thoracotomy approach. Ministernotomy for AVR has been associated with significant reduction in intraoperative blood loss compared to counterparts undergoing median sternotomy, though transfusion requirements were unchanged (262). This same prospective randomized study also reported no difference in respiratory function between the two groups, which was supported by results of another prospective RCT comparing outcomes in patients undergoing AVR with partial upper sternotomy vs. median sternotomy (263). This study also found the minimally invasive approach did not affect neurological outcomes or myocardial protection. In contrast, a separate randomized trial showed reduced transfusion requirement, shorter ventilation times, greater sternal stability, improved respiratory function, and earlier extubation and hospital discharge with ministernotomy AVR compared to median sternotomy approach (264).

Off-pump cardiac surgery has been attributed to reduced postoperative SIRS, but operative trauma, regional ischemia/reperfusion injury, and endotoxin release induced even in the absence of CPB and aortic cross-clamping may contribute to postoperative biological derangements and clinical morbidity. The endothelium has been increasingly implicated in multiorgan dysfunction following cardiac surgery, particularly in relation to hemostasis and oxidative stress. In a randomized clinical trial, patients undergoing off-pump cardiac surgery experienced reduction in systemic inflammatory response as measured by decreased plasma TNF-α, IL-10, myeloperoxidase, but avoiding CPB and aortic cross-clamping did not alter circulating levels of endothelial adhesion molecules (265). Differences between on-pump and off-pump cardiac surgery in this context have been limited to the final steps of the operations and early hours thereafter, suggesting that global surgical trauma may play a more important role in activation of systemic inflammatory and coagulation-fibrinolytic pathways (266). Complement activation and release of IL-8 is dependent on extracorporeal CPB circuit, while release of products of endothelial and leukocyte activation are temporally similar but decreased in magnitude in off-pump cardiac surgery. A procoagulant state and no rise in anti-inflammatory IL-10 following off-pump cardiac surgery may offset other benefits. Neurocognitive decline and pulmonary function outcomes following off-pump cardiac surgery are variable (267).

### 3.2 Pharmacologic strategies

#### 3.2.1 Aprotinin

Serine proteases comprise a large portion of effector proteins downstream of pro-inflammatory cytokines, complement activation, and hemolytic cascades. As these proteins amplify the inflammatory reaction, serine protease inhibitors have been investigated as potential therapies to mitigate excessive inflammation. Aprotinin is one such inhibitor and is perhaps one of the most well-studied. Aprotinin dually functions as an inhibitor of thrombin generation via the intrinsic pathway and has also been shown to preserve cellular junctions and reduce myocardial edema following cardioplegia and regional ischemia (268, 269). Its use has been previously associated with reduced intraoperative blood loss, while higher dosages may suppress the inflammatory response (270–272). These effects include attenuation of platelet activation, maintenance of platelet function, decreased complement and leukocyte activation, inhibition of kallikrein production, inhibition of endogenous cytokine-mediated NOS induction, inhibition of upregulation of adhesion molecules, and reduced release of several pro-inflammatory mediators (TNF-α, IL-6, and IL-8) (9, 150, 273–278). High-dose aprotinin has been associated with reduced post-CPB inflammation, myocyte damage, myocardial ischemia, and hospital length of stay in high-risk patients (7, 279, 280). High-dose aprotinin has also been associated with increased pro-inflammatory 8-isoprostane levels in the lungs relative to plasma levels. This effect disappeared with low-dose aprotinin, suggesting its action varies in a dose-dependent manner. The effect of high-dose aprotinin in decreasing circulating 8-isoprostane as estimated by lung passage (based on blood sampled from pulmonary and radial arteries) may signify a shift toward an anti-inflammatory milieu (281). Aprotinin may also decrease postoperative pulmonary and cerebral injury. Initial concerns of lower graft patency and renal dysfunction with aprotinin appear unfounded (7, 282). A meta-analysis revealed aprotinin reduced surgical blood loss, blood transfusion requirement, and need for redo thoracotomy. It also decreased perioperative mortality almost twofold without an associated increase in risk of myocardial infarction (270). A large, international, multi-institutional prospective study comparatively assessing the safety profile of aprotinin and lysine analogs (aminocaproic acid and tranexamic acid) use in patients presenting for coronary artery bypass surgery found that patients administered aprotinin had doubled risk of renal failure requiring dialysis, 55% increase in risk of myocardial infarction or heart failure, 181% increase in risk of stroke or encephalopathy, and increased risk of mortality. Neither of lysine analogs studied was associated with increased risk of cardiac, renal, or cerebral adverse events (283) and reduction in blood loss during surgery was similar for all three drugs. The BART study, a blinded RCT comparing aprotinin and lysine analogs in patients undergoing high-risk cardiac operations had to be prematurely terminated due to increased mortality associated with aprotinin, leading to suspended use of aprotinin in these patients (284). Subsequent initiatives that revisited limitations of these studies resulted in resumed use of aprotinin in select patients in both Canada and the European Union, while use of aprotinin in USA is still restricted.

#### 3.2.2 Phosphodiesterase inhibitors

Pentoxifylline is a nonspecific phosphodiesterase inhibitor with various anti-inflammatory effects, including attenuation of TNF-α and endotoxin release, cytokine synthesis, pulmonary leukocyte sequestration and vascular resistance, and reduction in indices of endothelial injury and permeability (285–289). Pentoxifylline was associated with decreased levels of pro-inflammatory cytokines (TNF-α and IL-6). Its use was also associated with improved left ventricular ejection fraction, decreased ICU length of stay, ventilation time, requirement of inotropic agents, and transfusion requirement (290).

Other phosphodiesterase inhibitors have been evaluated in the context of maximizing splanchnic perfusion as a strategy to attenuate excessive inflammation associated with CPB. Milrinone has been associated with reduction in venous and hepatic endotoxin levels, decrease in gastric intramucosal pH, and decreased IL-6 levels postoperatively in otherwise healthy patients undergoing cardiac operations (291, 292).

#### 3.2.3 Corticosteroids

Steroids have potent anti-inflammatory effects, and the mechanisms by which they exert their effects are multifactorial. Preoperative administration of glucocorticoids has been shown to attenuate endotoxin release and complement activation in response to CPB (293–295). Methylprednisolone was associated with decreased levels of postoperative pro-inflammatory mediators IL-6, IL-8, and TNF-α along with increased levels of anti-inflammatory IL-10 and IL-1ra (120, 121, 275, 296–300). Corticosteroids blunted the activation of leukocytes, upregulation of neutrophil adhesion molecules, and sequestration of neutrophils in the pulmonary parenchyma and vasculature (275, 294, 301, 302). Combination treatment of patients with methylprednisolone and aprotinin resulted in improved postoperative indices of cardiovascular, pulmonary, hemostatic, and renal function (303). Low-dose aprotinin had similar effect as methylprednisolone in blunting TNF-α release and neutrophil integrin CD11b upregulation (275). Another study showed methylprednisolone pretreatment was associated with improved cardiac performance and decreased bronchial inflammation post-CPB (275, 300). Short course of methylprednisolone has been shown to reduce incidence of postoperative atrial fibrillation (304). Low-dose methylprednisolone in pump priming solutions attenuated degree of myocardial damage (305). Preoperative plus pre-CPB administration may be superior to pre-CPB administration alone. Methylprednisolone prophylaxis was associated with lower levels of neuron-specific enolase, a biomarker for neuronal damage, suggesting it may be useful in reducing post-CPB cerebral injury (296). Benefits of corticosteroid use for reducing pulmonary inflammation, endotoxemia, and complemented activation are still disputed given the results of more recent RCTs, and differences in dosing regimen, formulation, and timing of administration may partially account for conflicting results. The SIRS trial showed no difference in risk of death or major morbidity between those randomized to receive methylprednisolone or placebo at 30 days postoperatively, while the most common adverse effects in both experimental arms were infectious or delirium-related (306). Overall, a clear benefit attributed to corticosteroid treatment in the setting of CPB remains to be demonstrated (293, 302, 306–310). The need for further studies investigating optimal dosage regimens, characterizing adverse events, and optimizing clinical outcomes cannot be overstated.

#### 3.2.4 Antioxidants and free radical scavengers

Myocardial antioxidant enzymes (including glutathione reductase, superoxide dismutase, and catalase) become activated in proportion to the degree of myocardial ischemia and reperfusion injury, but host antioxidants may become depleted after CPB (311–313). When ROS production exceeds host defense scavenging capacity, cellular injury results (314, 315). Increased preoperative total plasma antioxidant status has been associated with decreased levels of lipid peroxidation, which is directly correlated with indices of myocardial injury (313).

Vitamin C and vitamin E levels decline intraoperatively and remain low over two days postoperatively (316). High-dose vitamin C is an effective scavenger of free radicals and has been associated with decreased membrane lipid peroxidation, indices of myocardial injury, improved hemodynamics, and shorter ICU and hospital length of stay (314, 317). High-dose vitamin E has been associated with decreased plasma hydrogen peroxide concentrations and decreased membrane lipid peroxidation after CPB (26, 314). Prophylactic coadministration of vitamin C, vitamin E, and n-PUFAs (eicosapentaenoic acid:docosahexaenoic acid ratio 1:2) has been associated with reduced incidence of postoperative atrial fibrillation (318).

Allopurinol inhibits xanthine oxidase, a pivotal generator of free radicals during reperfusion injury. Some studies have demonstrated allopurinol reduced myocardial formation of cytotoxic free radicals, decreased myocardial injury, and improved myocardial recovery following CPB (315, 319–322). Other studies showed conflicting results, showing no benefit in myocardial injury or function with allopurinol alone (319, 323, 324). Preoperative supplementation of allopurinol in combination with vitamin C and vitamin E reduced cardiovascular dysfunction in both stable and unstable patients undergoing CABG, with unstable patients sustained lesser degree of myocardial injury and lower incidence of perioperative myocardial infarction (325). Results of other studies, however, have refuted effects of vitamin C and vitamin E supplementation on myocardial injury (326). Use of allopurinol in neonates to improve neurodevelopment following cardiac operations for congenital heart disease is an active area of research as it has already demonstrated benefit in infants with hypoplastic left heart syndrome (327, 328).

Preoperative or intraoperative administration of high-dose N-acetylcysteine, another scavenger of free radicals has been shown to reduced neutrophil oxidative burst response and elastase activity (329–331). N-acetylcysteine improved oxygenation and lung mechanics in patients with known acute lung injury, though no change in rates of progression to acute respiratory distress syndrome was noted (332). Clinical outcomes were not significantly affected with regard to mortality, myocardial infarction, bleeding, transfusion requirements, intubation time, and hospital length of stay (333). Low-dose N-acetylcysteine as an adjunct to cardioplegia reduced myocardial oxidative stress in patients undergoing CABG (334). Modified N-acetylcysteine via preparation with activated carbons to create sustained-release microcapsules demonstrated greater cardioprotection than N-acetylcysteine alone in a rat model of myocardial ischemia-reperfusion (335). In another rat model of CPB, N-acetylcysteine was shown to ameliorate CPB-associated intestinal injury via reduction in inflammation and oxidative stress as measured by decreased levels of intestinal malondialdehyde, TNF-α, IL-6, and serum diamine oxidase (336). A number of studies have recently shown N-acetylcysteine to improve pulmonary, hepatic, and renal outcomes in patients undergoing CPB with and without preexisting pulmonary and renal insufficiency (337–342).

Mannitol pretreatment has been associated with decreased myocardial formation of cytotoxic free radicals following CPB (315).

Post-CPB endothelial dysfunction is in large part mediated by ROS. In this way, free-radical scavengers, antioxidants, and iron chelators represent a potential therapeutic adjunct to mitigate deleterious effects of CPB-associated inflammation.

#### 3.2.5 Monoclonal antibodies, complement inhibition, and inhibition of endothelial cell activation

Another approach for decreasing contact activation and downstream inflammation may be utilizing endogenous soluble complement inhibitors. An RCT investigating a monoclonal antibody to human C5 demonstrated its efficacy and safety in the setting of CPB. Inhibition of synthesis of mediators in complement activation and formation of adhesion molecules in a dose-dependent fashion clinically translated to a reduction in coagulopathy, myocardial injury, and postoperative neurocognitive deficits (343). Compstatin, a peptide inhibitor of complement, completely inhibited heparin-protamine-induced complement activation *in vivo* in non-human primates without associated adverse events (344).

Other promising strategies for complement inhibition therapy include C1 inhibitor, recombinant soluble inhibitor-1 or soluble complement receptor 1, monoclonal antibodies to C3 and C5a, neutrophil elastase inhibitor, membrane-bound regulators of complement, and attenuation of complement receptor-3-mediated adhesion of inflammatory cells to vascular endothelium (7). In unstable patients with acute myocardial infarction undergoing emergency CABG, administration of C1 esterase inhibitor effectively limited complement activation and reduced myocardial ischemia-reperfusion injury as measured by significant reduction in cardiac troponin I. Its use was associated with improved cardiac function and hemodynamic performance without an impact on early mortality (345, 346). Soluble human complement receptor 1 effectively inhibited complement activation during CPB and significantly decreased mortality and myocardial infarction in male patients (347). Neutrophil elastase inhibitor, sivelestat, reduced levels of neutrophil elastase, IL-6, and IL-8 while also attenuating the pattern of physiological deterioration of gas exchange as measured by relative effect on alveolar-arterial oxygen index (348).

C5 complement inhibitor, pexelizumab, may offer mortality benefit. Results of an RCT enrolling over 3,000 patients showed a significant risk reduction of death or postoperative myocardial infarction within 30 days postoperatively in patients undergoing CABG with or without valve surgery. However, the study was not powered to detection reduction in mortality alone (349). These results were reproduced in a more recent RCT. Additionally, an exploratory analysis showed a significant mortality benefit in high-risk patients (350).

Selective inhibition of vascular endothelial activation may reduce deleterious effects of uncontrolled inflammation. Adhesion molecular blockade may interfere with adherence within 24 h following CPB, thereby preventing neutrophil-mediated widespread organ damage. Blockade of neutrophil and endothelial selectin molecules resulted in notable attenuation of cerebral injury in an animal model of CPB and DHCA, while inhibition of neutrophil adhesion markedly decreased pulmonary injury in a swine model of CPB (351, 352). A caveat to adhesion molecule blockade is increased susceptibility to infection due to impaired neutrophil demargination and recruitment to sites of infection (353). Strategies to prevent nuclear localization of transcriptional activator NF*κ*B, a key mediator of pro-inflammatory signaling, have also showed promise but studies demonstrating efficacy and safety are pending (151).

#### 3.2.6 Cyclooxygenase inhibitors

Constitutive cyclooxygenase 1 (COX-1) and its inducible isoform, cyclooxygenase 2 (COX-2), are sensible targets for modulating immune activation in response to CPB. Antiplatelet agents, including inhibitors of COX-1 and COX-2, prevent platelet aggregation. Acetylsalicylic acid, or aspirin (ASA), is one of the most commonly prescribed medications for the prevention of cardiovascular diseases. It irreversibly acetylates a serine residue of COX-1, thereby preventing release of thromboxane A2 and its downstream effects on platelet aggregation. The beneficial effects of ASA are not confined to platelet aggregation, as other mechanisms including attenuating inflammation, reducing oxidative stress, inhibiting prostaglandin formation, and inhibition of thromboxane-mediated vasoconstriction may be modulated for clinical benefit. Continued ASA treatment until the time of CABG has been shown to reduce inflammation as demonstrated by lower levels of plasma high-sensitivity CRP at all time points, though no change in cytokines was observed (354). Aspirin and clopidogrel in combination with aprotinin did not significantly affect clinical outcomes (355).

## 4 Limitations and conclusion

Over the past several decades since modern extracorporeal circulation was first conceived of by Gibbon, strategies for controlling CPB-associated inflammation had some success but have fallen short of controlling SIRS. Surface modification of the extracorporeal circuit, technical advances including control of flow dynamics in the CPB circuit, and mechanical refinements in pumps, oxygenators, tubing, filters, and other material components of the CPB circuit have reduced adverse sequelae and shown clinical benefit. Initial studies completed several decades prior investigating strategies that reduced circulating interleukins and other pro-inflammatory mediators showed limited translational benefit or showed contradictory results; more recent studies investigating these strategies are few as new technologies and therapies have emerged. Appropriate application of adsorptive blood purification techniques or use of immunomodulatory pharmacologics to mitigate hyperinflammatory states following CPB still remains uncertain given inconclusive efficacy and safety results from several studies. Though initially promising, aprotinin has been associated with a significant adverse event profile in target populations reported in several large studies, leading to restricted use in several countries. Postoperative SIRS may delay diagnosis of sepsis and septic shock following cardiac surgery, particularly in high-risk patients. The Sequential Organ Failure Assessment (SOFA) score may be more sensitive for predicting physiologic effects of infection, while Sepsis-3 criteria may be a useful tool for early identification and management of sepsis in patients following cardiac surgery (356). Overall, improving biocompatibility of the CPB circuit and more minimally invasive techniques may lead to improved myocardial preservation. Investigations into pharmacological adjuncts to more specifically and effectively attenuate inflammation continue. A multimodal approach incorporating technical, circuit-specific, and pharmacologic strategies will likely yield maximal clinical benefit.
